# Electronic conduction during the formation stages of a single-molecule junction

**DOI:** 10.3762/bjnano.9.138

**Published:** 2018-05-17

**Authors:** Atindra Nath Pal, Tal Klein, Ayelet Vilan, Oren Tal

**Affiliations:** 1 Department of Chemical and Biological Physics, Weizmann Institute of Science, Rehovot 7610001, Israel,; 2 Department of Condensed Matter Physics and Material Sciences, S. N. Bose National Centre for Basic Sciences, Block JD, Sector III, Salt Lake, Kolkata 700 106, India

**Keywords:** break junction, electron–vibration interactions, electronic transport, inelastic electron tunneling spectroscopy, molecular junction, molecular vibration, quantum interference, shot noise

## Abstract

Single-molecule junctions are versatile test beds for electronic transport at the atomic scale. However, not much is known about the early formation steps of such junctions. Here, we study the electronic transport properties of premature junction configurations before the realization of a single-molecule bridge based on vanadocene molecules and silver electrodes. With the aid of conductance measurements, inelastic electron spectroscopy and shot noise analysis, we identify the formation of a single-molecule junction in parallel to a single-atom junction and examine the interplay between these two conductance pathways. Furthermore, the role of this structure in the formation of single-molecule junctions is studied. Our findings reveal the conductance and structural properties of premature molecular junction configurations and uncover the different scenarios in which a single-molecule junction is formed. Future control over such processes may pave the way for directed formation of preferred junction structures.

## Introduction

**Figure 1:**
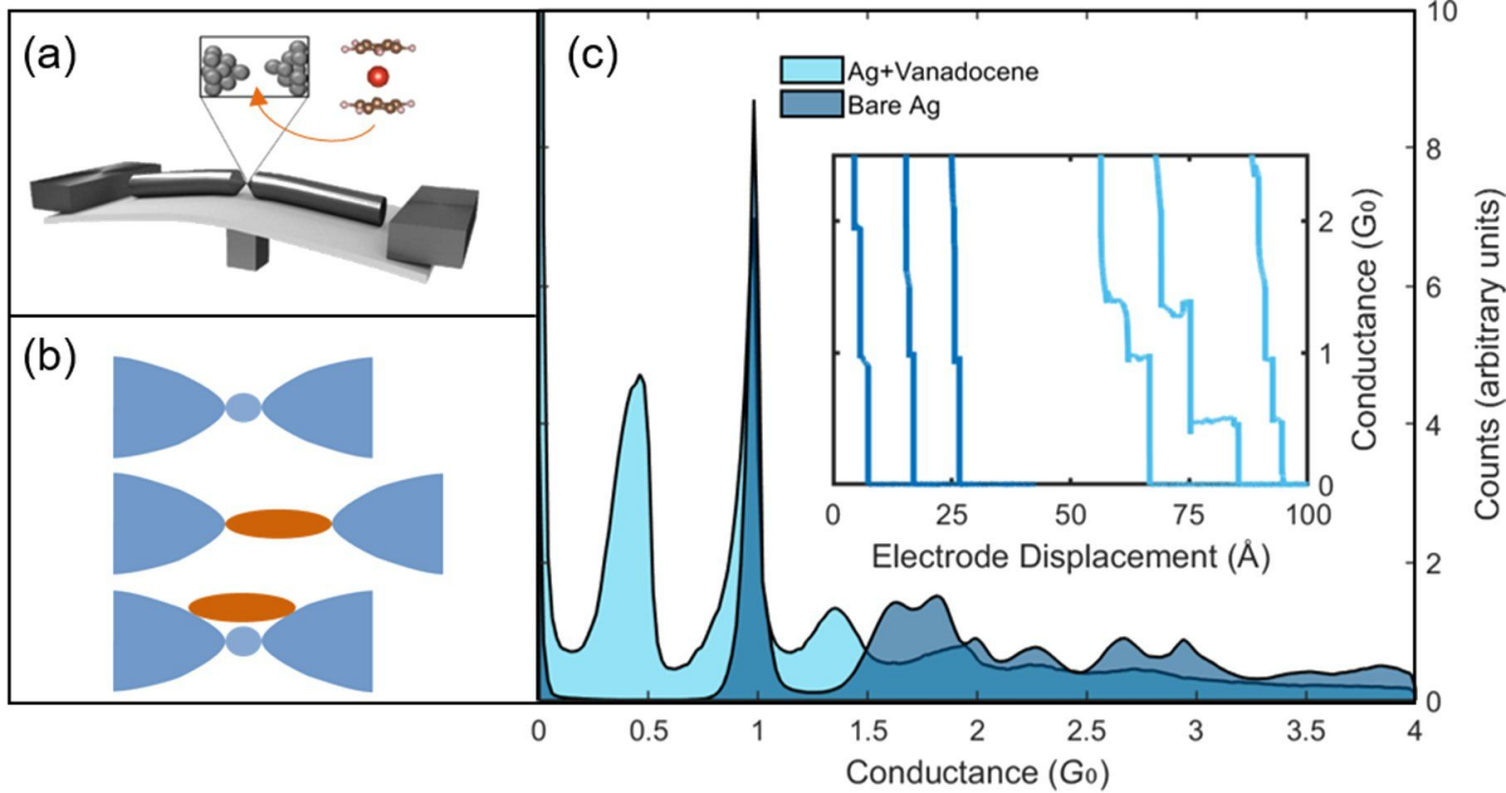
(a) Schematic of a mechanically controllable break junction device. Inset: Schematic of a vanadocene molecule and a broken junction. (b) Schematic illustration of a single-atom junction (top), a single-molecule junction (middle), and parallel single-atom and single-molecule junctions (bottom). (c) Conductance histograms, indicating the most probable conductance of the Ag junction during the final stages of junction elongation before (blue) and after (light blue) the introduction of vanadocene. Both histograms were taken at a bias voltage of 100 mV. Each histogram is composed from 10,000 conductance traces. Inset: Examples for conductance vs electrode displacement traces measured while elongating the atomic (blue, left) and molecular (light blue, right) junctions. The traces are shifted for clarity.

Single-molecule junctions serve as a versatile atomic-scale laboratory for quantum electronic transport [1–2]. The formation of such molecular junctions, where a molecule is suspended as a bridge between two metallic electrodes, was greatly facilitated by the development of the mechanically controllable break junction technique [3]. In a break junction (Figure 1a), molecules are introduced to a metallic junction while it is stretched. As a result, the junction is thinned to an atomic scale contact and eventually it breaks to form two electrode apexes. In some cases, a molecule is trapped between the electrodes, forming a single-molecule junction (Figure 1b). For more than two decades, the electronic transport properties of single-molecule junctions based on organic or organometallic molecules were explored in numerous of studies [1–2]. However, information about the conductance properties of such molecular junctions at their early stages of formation is missing. In fact, the conductance signature of premature configurations of molecular junctions (before breaking the atomic scale contact) was studied only for diatomic molecules [4–6]. Here, we use conductance measurements, inelastic electron spectroscopy, and shot noise analysis to identify the formation of a single-molecule junction based on an organometallic vanadocene molecule (Figure 1, inset) in parallel to a single atom silver (Ag) junction. The interplay between the two conductance pathways via the molecular bridge and across the metallic one is characterized in terms of additive independent conductance pathways, quantum interference between the two pathways, and deformed electronic structure by the presence of molecules. Finally, we reveal the different scenarios of structural evolution from premature junction configurations towards the formation of a typical metal–molecule–metal junction, bringing to light the early steps of single-molecule junction formation.

## Experimental

We use the mechanically controllable break junction (MCBJ) technique [3] to fabricate atomic-sized junctions (Figure 1a,b). A Ag wire (99.997%, 0.1 mm, Alfa Aesar) with a notch in its center is fixed onto a flexible substrate. This structure is placed in a vacuum chamber and cooled to 4.2 K. To form an atomic scale junction, the substrate is pushed and bent at its center by a piezoelectric element. As a result, the two sides of the notch are pulled apart and the cross section of the notch is gradually reduced until a single-atom junction is formed between the wire segments [7]. Further bending leads to breaking of the wire and the formation of two freshly exposed electrode apexes in cryogenic vacuum conditions. Molecular junctions are prepared by sublimating vanadocene molecules (95%, Buchem, further purified in situ), from a locally heated molecular source towards the metallic junction, while repeatedly breaking and reforming the junction between the two electrodes to study molecular junctions with different local structure [8].

## Results and Discussion

First, the conductance of the bare Ag junction was recorded as a function of the relative displacement of the electrodes' apexes. Figure 1c (inset) presents examples of such conductance traces (blue, left). During the breaking process, the conductance is reduced in a sequence of abrupt steps as the number of atoms in the junction’s cross section decreases [7]. The last plateau at ≈1 *G*_0_ is associated with a single-atom junction [9–10]. Further increase in the interelectrode separation leads to junction breakage and an abrupt conductance drop to the tunneling transport regime. When vanadocene molecules are introduced, additional conductance plateaus appear below 1 *G*_0_, indicating the formation of a metal–molecule–metal junction after the Ag contact is broken. Interestingly, the introduction of molecules into the metal junction also yields new plateaus at ≈1.3 *G*_0_, which is above the typical conductance of a single-atom junction. These conductance plateaus clearly indicate the presence of molecules, since they cannot be found for bare Ag junctions.

The exact details of the mentioned conductance plateaus can be different for different conductance traces due to variations in the atomic scale structure of repeatedly formed junctions. To collect statistical information about the most probable conductance of the bare Ag and Ag–vanadocene junctions, we constructed conductance histograms based on thousands of traces. Figure 1c presents two conductance histograms taken before and after the introduction of vanadocene molecules. The histogram for bare Ag junctions (blue) shows a conductance peak at ≈1 *G*_0_, which is related to single-atom junctions, and a tail at low conductance due to electron tunneling after the junctions are broken. The introduction of vanadocene molecules (light blue histogram) yields another conductance peak at ≈0.5 *G*_0_, which is lower than the typical conductance of the Ag atomic junction, as expected when a molecule bridges two metallic electrodes [8]. However, the emergence of another peak at ≈1.3 *G*_0_ in response to the introduction of molecules suggests the formation of a molecular junction in parallel to a metal atomic junction or, alternatively, the formation of a metal junction (e.g., a diatomic contact) with modified conduction due to adsorbed molecules. Individual traces (e.g., Figure 1c, inset) show that the 1.3 *G*_0_ plateaus are recorded before the 1 *G*_0_ plateaus. Namely, the associated structure with the 1.3 *G*_0_ plateaus is formed before the single-atom contact breaks. Thus, a third option of several molecules suspended between the electrodes that contribute a total conductance of 1.3 *G*_0_ is highly unlikely.

Inelastic electron spectroscopy [11–14] can offer valuable information about the structure of the combined metallic and molecular junctions. When an applied voltage (*V*) across a molecular junction exceeds the energy (in eV) of a certain molecular vibration mode, some of the transmitted electrons lose energy to excite the vibration mode. For weak electron–vibration coupling in the off-resonance regime, these electrons are inelastically scattered forward or backward, yielding a step up or down in the conductance (conductance enhancement or suppression), respectively [13]. The step is located at a voltage equivalent to the vibration energy and its height is equal to the inelastic conductance contribution to the overall conductance across the junction.

**Figure 2:**
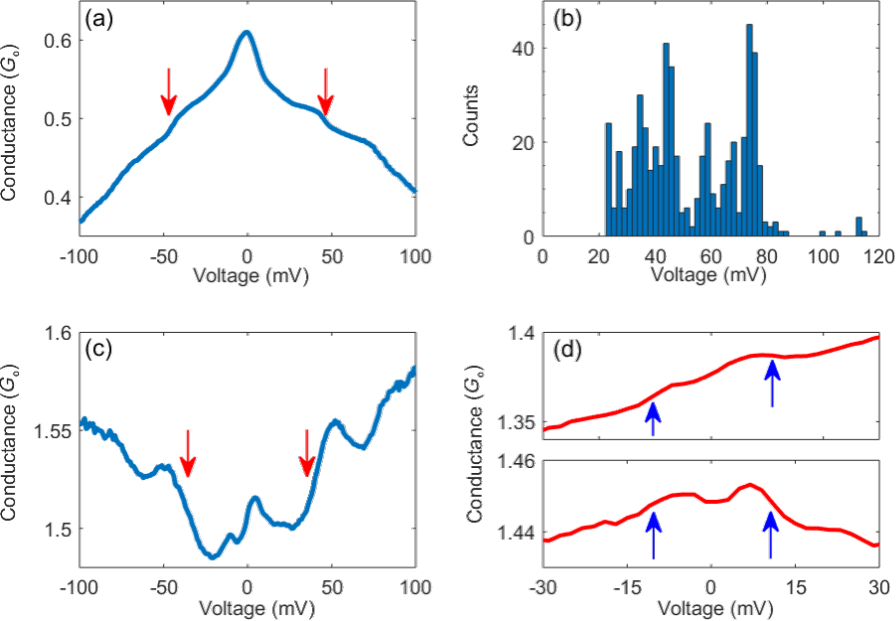
(a) Differential conductance vs applied voltage (d*I*/d*V* vs *V*) spectra measured at ≈0.6 *G*_0_ zero-voltage conductance after the introduction of vanadocene to the Ag junction. The steps in the conductance curve that are considered in the text are marked by arrows. Additional steps at higher voltage (not marked) can be seen as well. (b) Histogram of the number of times that a step feature at a certain applied voltage appears in d*I*/d*V* vs *V* spectra measured on different realizations of Ag–vanadocene junctions (544 spectra were analyzed in the context of step voltage). (c) Similar to (a) at ≈1.5 *G*_0_ zero-voltage conductance. Note that steps down (up) in the conductance are a consequence of vibration interaction with a conduction channel that contributes more (less) than 0.5 *G*_0_ [13]. (d) d*I*/d*V* vs *V* spectra taken after the introduction of molecules at ≈1.38 *G*_0_ (top) and ≈1.45 *G*_0_ (bottom) zero-voltage conductance. The steps in the conductance appear below 25mV and are associated with activation of metal phonon modes [15].

Figure 2a shows a differential conductance curve (d*I*/d*V* vs *V*) taken across a Ag–vanadocene junction with a zero-voltage conductance of ≈0.6 *G*_0_. The observed conductance steps at 45 ± 1 mV take place at higher voltage than expected for conductance steps due to Ag phonon excitations (<25 mV) [15], and are ascribed to activation of a vibration mode in the molecular junction [13]. The overall distribution of steps as a function of voltage is seen in Figure 2b. The observation of steps at the same value of positive and negative voltage, and the repeatable appearance of steps at specific voltage values, as seen in Figure 2b, support the vibrational origin of these steps. Remarkably, as seen in Figure 2c, also junctions with zero-voltage conductance of ≈1.5 *G*_0_ show conductance steps at 43 ± 1 mV, indicating a finite conductance across a molecule. These findings reveal the existence of a conducting molecular junction in parallel to a metallic junction. In this structure, the onset of the inelastic contribution to the conductance at 43 ± 1 mV stems from electronic transport via the molecular junction, while the overall conductance is the outcome of the parallel molecular and metallic pathways. The zero-voltage feature observed in Figure 2a,c does not allow a clear identification of phonon-induced steps in the conductance via the metallic bridge, which are expected below 25 mV. However, in some cases, this feature is suppressed and conductance steps can be seen at ≈10 mV for junctions with zero-voltage conductance around 1.3 *G*_0_ (Figure 2d), perhaps due to inelastic conductance via the metallic junction.

The vibration step height can be different for different junction realizations, since electron–vibration interaction is not always efficient. The presented examples represent the cases in which maximal step height was found for junctions with conductance below and above 1 *G*_0_ (181 cases were examined). The similar step height (0.05 ± 0.01 *G*_0_) in both cases infers a similar inelastic conductance across the molecule, both for the single-molecule junction and for the single-molecule junction in parallel to a metal junction. If the ratio between inelastic and elastic conductance across the molecular bridge is roughly identical in both cases (e.g., due to similar electron–vibration coupling), then the total conductance via the molecular bridge should be similar for the two examined cases. This suggests that for the junction configuration with conductance of ≈1.5 *G*_0_, the conductance through the molecular pathway is roughly 0.6 *G*_0_, as in the case of the single-molecule junction, while the conductance via the metallic pathway is about 0.9 *G*_0_. Thus, the studied examples support the existence of a conducting single-molecule junction in parallel to a single-atom Ag junction, with additive conductance.

The direction of the vibration-induced steps in the conductance can provide complementary information about the structure of the combined metallic and molecular junctions. Steps down (up) in the conductance are expected when a conduction channel with a contribution of more (less) than 0.5 *G*_0_ interacts with a vibration mode [13]. This “0.5 crossover” is an outcome of competing backward and forward inelastic electron scattering contributions, where the former contribution is dominant at low conductance (e.g., tunneling transport via molecular junctions [11]) and the latter one is dominant at high conductance (e.g., transport at the contact regime via atomic junctions [12]). Focusing on the examined cases, the steps down in Figure 2a are expected for higher conductance than 0.5 *G*_0_ as indeed observed. In contrast, the steps up in Figure 2c imply the interaction of a molecular vibration mode with a secondary conduction channel across a molecular junction with conductance contribution of less than 0.5 *G*_0_. Note that this contribution is lower than estimated by the step height. Although the difference can stem from another conduction channel via the molecular junction that does not interact with vibration modes, one should bear in mind that both analysis methods provide only a rough estimation for the conductance distribution. Coming back to the step direction analysis, the remaining conductance contribution (which is 1 *G*_0_ or slightly higher) is transmitted via one or more conduction channels that do not interact with molecular vibrations. Overall, the analysis of the step height and direction support a secondary conductance contribution via a conducting molecular junction and a dominant conductance contribution given by a neighboring single-atom junction.

Using shot noise measurements, we can gain more reliable information about the distribution of conduction channels across the parallel atomic and molecular junctions. Specifically, shot noise analysis can provide information about quantum interference in electronic transport through the atomic and molecular pathways. Current shot noise is generated since each injected electron into the junction is either transmitted or scattered back, leading to time-dependent current fluctuations [16]. In the framework of Landauer formalism [17–18], shot noise depends on the number of conduction channels, *i*, across the examined junction and the transmission probability of each channel, τ*_i_*. When an applied voltage across a quantum conductor such as an atomic or molecular junction satisfies eV >> *kT*, the generated shot noise is given by *S* = 2e*IF* where e is the electron charge, *I* is the current, and *F* = ∑*_i_* τ*_i_* (1 − τ*_i_*) / ∑*_i_* τ*_i_* is the Fano factor that can be determined by the noise dependence on applied current bias [19].

The two independent equations for conductance and shot noise analytically provide the transmission probabilities for junctions with up to two conduction channels. Since the number of conduction channels across the examined junctions is unknown, the transmission probability of each channel is then determined numerically with a limited accuracy [20]. We start by assuming a certain number of channels and we find all possible transmission probabilities (with 1% resolution) that give the measured shot noise and conductance in a given junction. The process is then repeated for a higher number of channels until the results converge. This conduction channel analysis was shown to be very efficient in identifying a variety of electronic transport properties of atomic-scale junctions [20–24].

**Figure 3:**
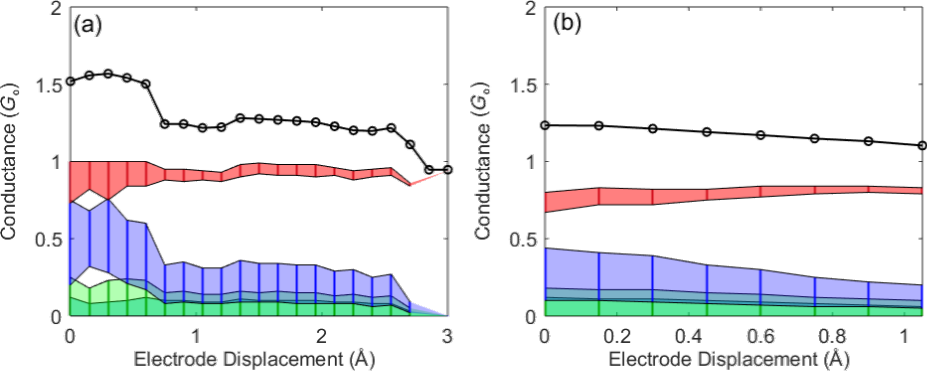
(a,b) Conductance traces measured for the Ag–vanadocene junction (black curve) and the experimentally resolved conductance contributions of the main channels based on conductance and shot noise analysis [20]. For junctions with conductance around 1.3 *G*_0_ only two main channels are always open. In this analysis we assume four spin-degenerate channels. However, repeating the analysis under the assumption of three and two channels gave similar results, indicating that the total conductance is probably dominated by only two channels. Note that spin-resolved channel analysis does not change the overall outcome of a major spin-degenerate contribution of 0.75–1 *G*_0_ and the rest is dominated by a secondary channel. The limited accuracy of the channel analysis stems from avoiding the a priori assumption of only two conduction channels.

Based on conductance and shot noise measurements, the contributions of the main conduction channels to the total conductance across the parallel metallic and molecular junctions were identified. Figure 3 shows two examples for the distribution of conduction channels for such junctions, which are characterized by a ≈1.3 *G*_0_ conductance plateau (black). In Figure 3a the total conductance is given by two main channels that contribute roughly 1 *G*_0_ and 0.3 *G*_0_, when the junction is stretched beyond electrode displacement of 0.7 Å. The conductance of a single-atom junction of Ag is typically 1 *G*_0_, dominated by a single conduction channel [9,20], and the conductance of the single-molecule junction after the breaking of the Ag junction is typically 0.3–0.6 *G*_0_, as seen in Figure 1c. Thus, the channel distribution of the examined junction seems to further support the scenario of a single Ag atom junction in parallel to a single-molecule junction, where the conductance of the two pathways is independent and additive. In 22 out of 32 junctions examined by shot noise analysis, the main conduction channel contributes about 1 *G*_0_, to the total ≈1.3 *G*_0_ conductance and the rest is given by a secondary channel. This channel distribution rules out significant conductance interference in the parallel metallic and molecular junctions, since any notable interference would lead to deviations from the trivial channel distribution of ≈1 *G*_0_ and ≈0.3 *G*_0_.

In contrast, Figure 3b exemplifies the typical channel distribution that was found for the remaining 10 examined cases. Here, the total conductance is given by two main channels, each with lower conductance contribution than 1 *G*_0_. The deviation from the trivial channel distribution can stem from distorted local electronic structure at the single-atom junction due to the presence of the molecular bridge or other nearby adsorbed molecules, such that the conductance of the metallic junction is suppressed [25–26]. The parallel molecular junction can also suppress the conduction via the atomic junction by stabilizing atomic structures that do not usually survive in bare metallic junctions [27] (e.g., allow elongated structures with a large inter atomic distance). Alternatively, quantum interference between the molecular and the atomic pathways can generate such nontrivial channel distribution. The first option (i.e., distortion of the local electronic structure) should also lead to asymmetric widening of the 1 *G*_0_ peak in the conductance histogram towards lower conductance, since the presence of adsorbed molecules may lower the conductance of the single-atom junction even in the absence of a parallel molecular bridge. Such an asymmetric widening is indeed observed for the 1 *G*_0_ peak in Figure 1c. So although we cannot rule out quantum interference in the examined cases, most likely the conductance suppression observed for the main channel results from the influence of the nearby adsorbed molecule(s) on the electronic structure of the single Ag atom junction.

**Figure 4:**
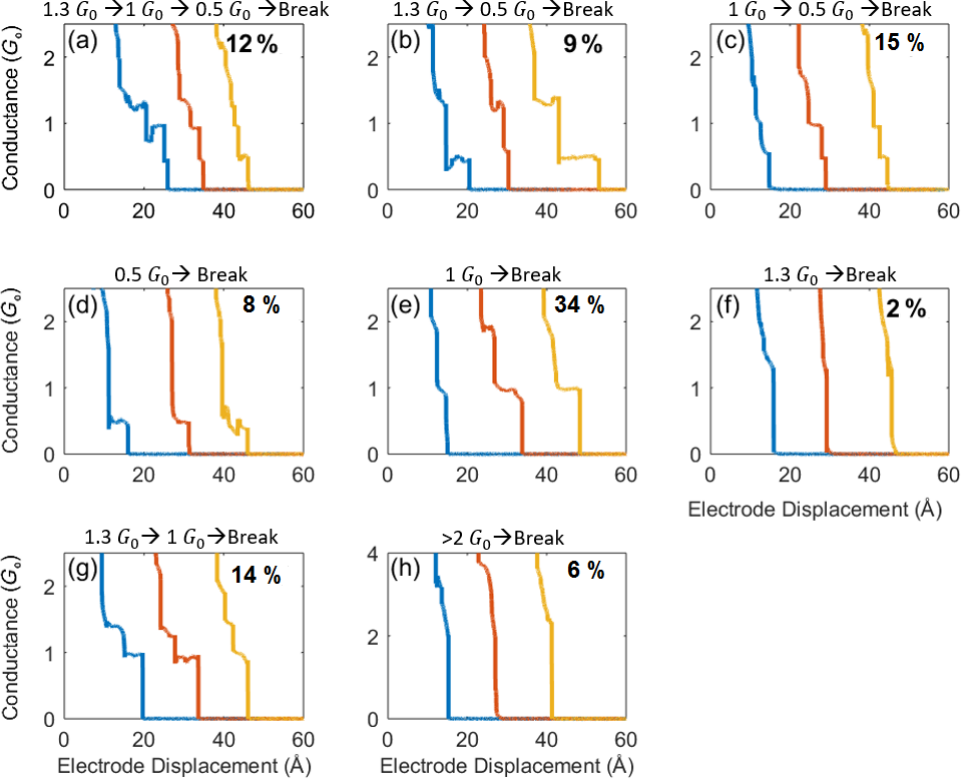
(a−h) Examples for sets of conductance traces taken during repeated junction breaking with different combinations of plateaus (an ensemble of 50,000 traces was analyzed). The different sets illustrate a variety of scenarios for the structural evolution of the Ag–vanadocene junction. The percentage of each set of traces is indicated. For the sake of simplicity we ignore molecular configurations with conductance around 10^−2^–10^−3^ *G*_0_.

To shed light on the different scenarios for the evolution of Ag–vanadocene junctions, we examine the occurrence probability of different sequences of junction configurations. For this task we consider the probability to find different combinations of plateaus at ≈1.3 *G*_0_, ≈1.0 *G*_0_, and ≈0.5 *G*_0_, which we associate with parallel junctions of a single metal atom and a single molecule, a single-atom junction, and a single-molecule junction, respectively (Figure 1b). Using a plateau-identification code, we tagged each plateau as 1.3 *G*_0_, 1 *G*_0_ or 0.5 *G*_0_, if it had at least 20 data points within a tolerance window of 1.7–1.2 *G*_0_, 1.15–0.8 *G*_0_ or 0.75–0.2 *G*_0_, respectively. The tolerance window is determined by the corresponding peak width in the conductance histogram (e.g., Figure 1c). The different scenarios are presented in Figure 4. About 44% of the junctions evolve to a single-molecule junction, albeit via different sequences of configurations (scenarios a–d). A bare atomic junction is formed merely in 34% of the cases (scenario e). The most probable scenario for the formation of a single-molecule junction does not involve the formation of a parallel junction configuration (scenario c), indicating that the parallel junction is not a necessary precursor configuration for the formation of the single-molecule junction. In fact only 9% of the junctions evolve from a parallel junction configuration to a single-molecule junction (scenario b). Alternative pathways towards a single-molecule junction may include the formation of a single-atom junction or a junction with more than a single atom in its narrowest cross section (with conductance of ≈1 *G*_0_ and >2 *G*_0_, respectively), that is followed by the insertion of a molecule into the junction (scenarios c and d, respectively). Finally, complete breaking of the parallel junction configuration is highly unlikely (2%, scenario f), probably since it involves simultaneous breaking of a single atom and a single-molecule junction. Instead, stretching the parallel junction configuration leads to the breaking of the atomic junction (scenario b), or alternatively, the breaking of the molecular junction (scenarios a and g), while the other junction is preserved.

## Conclusion

The early stages of single-molecule junction formation are analyzed in this work with the aid of conductance vs displacement measurements in a break junction setup, inelastic electron spectroscopy and shot noise analysis. We establish the existence of parallel molecular and atomic junctions, where a single vanadocene junction is constructed in parallel to a single Ag atom junction between two Ag electrodes. The combination of inelastic electron spectroscopy and shot noise analysis allows us not only to address the total conductance of the parallel junctions as in standard conductance measurements but also to estimate the relative contribution of the two pathways to the total conductance. We found that in about two thirds of the examined cases the conductance contributions of the neighboring junctions are independent and additive. However, in about one third of the cases, the contribution of each one of the two main conduction channels is lower than the typical conductance of a single-atom junction. This deviation can be explained by molecule-induced distorted local electronic structure or by quantum interference. Finally, the formation of a single-molecule junction from premature junction structures is analyzed, revealing a rich set of scenarios for structural evolution towards the final realization of a single-molecule bridge, not necessarily via the parallel junction configuration. Hopefully, further study of premature junction structures and their evolution towards single-molecule junctions will pave the way for directed formation of desired molecular junction structures by controlling their structural evolution.

## References

[1] Cuevas J C, Scheer E (2017). Molecular electronics: an introduction to theory and experiment.

[2] Aradhya S V, Venkataraman L (2013). Nat Nanotechnol.

[3] Muller C J, van Ruitenbeek J M, de Jongh L J (1992). Phys Rev Lett.

[4] Aradhya S V, Frei M, Halbritter A, Venkataraman L (2013). ACS Nano.

[5] Balogh Z, Visontai D, Makk P, Gillemot K, Oroszlány L, Pósa L, Lambert C (2014). Nanoscale.

[6] Balogh Z, Makk P, Halbritter A (2015). Beilstein J Nanotechnol.

[7] Agraït N, Yeyati A L, van Ruitenbeek J M (2003). Phys Rep.

[8] Yelin T, Korytar R, Sukenik N, Vardimon R, Kumar B, Nuckolls C, Evers F, Tal O (2016). Nat Mater.

[9] Ludoph B, van Ruitenbeek J M (2000). Phys Rev.

[10] Rodrigues V, Bettini J, Rocha A R, Rego L G C, Ugarte D (2002). Phys Rev B.

[11] Stipe B C, Rezaei M A, Ho W (1998). Science.

[12] Agraït N, Untiedt C, Rubio-Bollinger G, Vieira S (2002). Phys Rev Lett.

[13] Tal O, Krieger M, Leerink B, van Ruitenbeek J M (2008). Phys Rev Lett.

[14] Kim Y, Pietsch T, Erbe A, Belzig W, Scheer E (2011). Nano Lett.

[15] Khotkevich A V, Yanson I K (2013). Atlas of Point Contact Spectra of Electron-Phonon Interactions in Metals.

[16] van den Brom H E, van Ruitenbeek J M (1999). Phys Rev Lett.

[17] Büttiker M, Imry Y, Landauer R, Pinhas S (1985). Phys Rev B.

[18] Büttiker M (1992). Phys Rev B.

[19] Blanter Ya M, Büttiker M (2000). Phys Rep.

[20] Vardimon R, Klionsky M, Tal O (2013). Phys Rev B.

[21] Ben-Zvi R, Vardimon R, Yelin T, Tal O (2013). ACS Nano.

[22] Vardimon R, Yelin T, Klionsky M, Sarkar S, Biller A, Kronik L, Tal O (2014). Nano Lett.

[23] Vardimon R, Klionsky M, Tal O (2015). Nano Lett.

[24] Stevens L A, Zolotavin P, Chen R, Natelson D (2016). J Phys: Condens Matter.

[25] Kiguchi M, Stadler R, Kristensen I S, Djukic D, van Ruitenbeek J M (2007). Phys Rev Lett.

[26] Landau A, Kronik L, Nitzan A (2008). J Comput Theor Nanosci.

[27] Huisman E H, Trouwborst M L, Bakker F L, de Boer B, van Wees B J, van der Molen S J (2008). Nano Lett.

